# Mapping Molecular Transitions in Barrett’s-Associated Oesophageal Adenocarcinoma via Multi-Omics Integration and Pathway Activity Modelling

**DOI:** 10.3390/cancers18132080

**Published:** 2026-06-26

**Authors:** Sabaoon Zeb, Pedro Henrique da Costa Avelar, Vicky Goh, Sophia Tsoka

**Affiliations:** 1 Department of Informatics, Faculty of Natural, Mathematical and Engineering Sciences, King’s College London, London WC2B 4BG, UK; sabaoon.zeb@kcl.ac.uk (S.Z.);; 2 Institute for Infocomm Research, Agency for Science Technology and Research (A*STAR), 1 Fusionopolis Way, #21-01 Connexis, Singapore 138632, Singapore; 3 Department of Cancer Imaging, School of Biomedical Engineering and Imaging Sciences, King’s College London, London SE1 7EH, UK; vicky.goh@kcl.ac.uk

**Keywords:** multi-omics integration, pathway activity inference, oesophageal adenocarcinoma, Barrett’s oesophagus, molecular heterogeneity, disease states

## Abstract

**Background/Objectives**: Interpretation of high-dimensional molecular profiling data requires computational strategies that go beyond single-layer analyses to resolve coordinated biological programs. Although multi-omics integration has advanced latent structure discovery, translating these cross-omics signals into interpretable, functionally meaningful molecular states remains a central challenge. **Methods**: Here, we present an interpretable, pathway-centric multi-omics integration framework that combines expression, DNA methylation, and copy-number alteration data to capture nonlinear cross-omics interactions and enable biologically grounded representations of molecular states. We apply this framework towards oesophageal adenocarcinoma, a malignancy that remains relatively underexplored in integrative multi-omics and systems-level analyses, despite its rising incidence and poor prognosis. **Results**: Using samples spanning Barrett’s oesophagus and oesophageal adenocarcinoma from the Oesophageal Cancer Clinical and Molecular Stratification consortium, we demonstrate the framework’s ability to resolve coordinated oncogenic, metabolic, immune, and cell-cycle programs that evolve across disease states. **Conclusions**: Together, this work establishes a scalable and interpretable computational strategy for pathway-based multi-omics integration, along with providing a generalisable approach for molecular state assignment in complex biological systems.

## Simple Summary

High-dimensional multi-omics data interpretation requires computational approaches that can integrate information across molecular layers and reveal coordinated biological processes. In this study, we develop a pathway-centric framework that integrates gene expression, DNA methylation, and copy-number alteration data using latent factor modelling and pathway activity inference. Applied to oesophageal cancer progression using data from the Oesophageal Cancer Clinical and Molecular Stratification consortium, the framework identifies distinct disease-associated molecular states driven by coordinated pathway activity associated with the transition from Barrett’s oesophagus to oesophageal adenocarcinoma. This approach enables biologically interpretable characterisation of molecular changes underlying cancer progression.

## 1. Introduction

The rapid growth of multi-layered high-dimensional omics data presents unparalleled opportunities but also significant analytical challenges in unveiling the layers of information within the tumour microenvironment (TME). While multi-omics datasets including transcriptomic, epigenomic, and genomic layers offer distinct perspectives on cellular states, the intricate, nonlinear interactions that characterise biological systems, modality-specific noise, and their varying scales make their integration challenging [1,2]. Although traditional single-omics analysis or feature-level approaches have provided significant insights over the years, they lack a holistic overview of the TME and often fail to capture cross-omics causal interactions underlying molecular states and disease progression. This restricts the ability to infer molecular programs or identify molecular states responsible for continuously evolving complex systems within the TME [3,4]. To overcome these constraints, computational frameworks that can detect functionally coherent molecular processes, integrate diverse omics layers, and generate interpretable and biologically grounded outputs are crucial.

Current multi-omics data integration strategies include early integration through feature concatenation, intermediate integration employing latent variable models (such as multi-omics factor analysis, iCluster, and canonical correlation analysis, among others), and late integration using ensemble learning or network-based techniques [5,6,7,8]. Intermediate integration enables the extraction of latent representations that encode shared and modality-specific signals across the omics layers, which ultimately reduces the dimensionality and uncovers coordinating molecular patterns underlying cellular states or the course of a disease [9]. Without presuming temporal ordering or clinical endpoints, integrative analysis offers great potential to investigate disease-linked molecular patterns and their interactions with phenotypic annotations.

A biologically significant link between multi-omics embeddings and functional interpretation is provided via pathway activity inference, which transforms high-dimensional molecular measurements into interpretable, sample-level functional scores. Latent signals can be translated into higher-order biological processes by mapping multi-omics-derived features onto functionally coherent units such as pathways [10,11]. This enables inference of the true biological mechanisms and intricate connections underlying the TME, particularly the progression of diseases. Using gene-level multi-omics data, tools such as PROGENy [12], Pathifier [10], decoupleR [13], DIOPTRA (Disease OPTimisation for biomaRker Analysis) [14] and PAAEs (pathway-activity autoencoders) [15] have provided estimation of the activity of biological pathways, hence enabling researchers to understand the behaviour of numerous disease-specific biological processes. In particular, by modelling nonlinear interactions across modalities, deep learning-based techniques like PAAE capture nonlinear cross-omics dependencies, enabling the construction of low-dimensional embeddings that summarises multi-layered discrete molecular states.

Here, we report an interpretable, pathway-centric multi-omics integration framework to capture complex nonlinear cross-omics interactions, identify functionally relevant pathways, and characterise coordinated molecular states across disease progression. We have applied this framework to matched multi-omics (including transcriptomic, epigenomic, and copy-number alterations) and clinical data from the Oesophageal Cancer Clinical and Molecular Stratification (OCCAMS) consortium [16]. The dataset consists of Barrett’s oesophagus (BO) and oesophageal adenocarcinoma (OAC) samples, a complex disease spectrum marked by heterogeneous, often shared, nonlinear alterations that add significant challenges to disease management by making conventional chemotherapy and targeted therapy trials often yield limited clinical benefit [17,18,19]. Globally, oesophageal cancer ranks as the 11th most commonly diagnosed cancer and the seventh leading cause of cancer death, with OAC incidence rising markedly across Europe, the United Kingdom, and North America over recent decades and five-year survival rates remaining below 20% despite advances in multimodal treatment [20,21,22]. Moreover, Barrett-related OAC remains relatively underexplored in integrative multi-omics and systems-level analyses. Therefore, our approach combines unsupervised machine learning for intermediate multi-omics integration, pathway activity inference, and interpretable modelling for identifying disease-associated molecular states. We demonstrate how multi-omics integration and pathway-level modelling can determine the complexity underlying disease progression, reveal mechanistic drivers, and enable the classification of molecular states. This approach provides a generalisable computational strategy for exploring the heterogeneity of underlying molecular states, the functional impact on TME, and the identification of pathways that can serve as mechanistic or prognostic anchors in various complex diseases.

## 2. Materials and Methods

### 2.1. Multi-Omics Dataset Preprocessing

Multi-omics data from the OCCAMS consortium [16] were used for this study, incorporating bulk RNA-sequencing (RNA-seq), DNA methylation arrays, and copy-number alterations (CNA) derived from whole-genome sequencing, along with matched clinical phenotype information across oesophageal adenocarcinoma (OAC), Barrett’s oesophagus (BO), and normal tissue. All data processing and analysis steps were conducted using R [23] (version 4.4.1; R Foundation for Statistical Computing, Vienna, Austria) and Python (version 3.12; Python Software Foundation, Wilmington, DE, USA) programming, with a specific focus on preparing the data for an integrative, pathway-level exploration using a multi-omics framework. The samples were cross-matched across all three omics layers, ensuring high-confidence integration and consistency in phenotype labelling. Quality control protocols were also applied independently to each type of omics data to eliminate outliers, handle missing data, and detect and correct batch effects. The overlap of samples across the three omics modalities from the OCCAMS cohort is summarised in Supplementary Figure S2.

#### 2.1.1. Bulk RNA-Seq Data Preprocessing and Batch Correction

We obtained transcript-per-million (TPM) normalised data for 391 samples across nine batches. Dimensionality reduction methods including principal component analysis (PCA) [24] and uniform manifold approximation and projection (UMAP) [25] (version 0.2.10.0) were used to assess technical variance, while limma [26] (version 3.58) and ComBat [27] (sva package, version 3.50) were applied for batch correction. To evaluate batch effects before and after batch correction, silhouette score analysis [28] (cluster package, version 2.1.8.1) was performed which exhibited minimal overall batch effects with some residual variance localised to a specific batch obtained in 2022. We then evaluated the impact of batch effects on biological signals or differentially expressed genes (DEGenes) obtained by differential expression analysis in which the Jaccard index [29] was calculated for pairwise comparison of batch–phenotype. With a lower Jaccard index, significant differences were identified, suggesting that batch effects had a minor impact on DEGenes according to the phenotype.

#### 2.1.2. Methylation Data Preprocessing and Annotation

The methylation data from OCCAMS were received in the raw methylation EPIC array format for approximately 865k CpG sites across 336 samples including 275 OAC, 29 BO and 32 normal samples. For the methylation data analysis, we used the minfi library in R. Using minfi [30] (version 1.48), the raw.idat files (EPIC array, 865k CpG sites, 336 samples) were imported into an RGChannelSet object, preprocessed with preprocessRaw, and converted to MethylSet, then RatioSet, and ultimately into a GenomicRatioSet (GRSet) with annotations. We then evaluated the intensity of the signals, which confirmed that the methylation data were of high quality. Finally, functional normalisation via preprocessFunnorm was used, incorporating noob background correction and quantile normalisation. The CpG probes were retained as CpG-level features throughout all downstream analyses. The probes were annotated to genes using the Illumina EPIC array manifest (v1.0 B5) through the UCSC_RefGene_Name field, preserving one-to-many CpG-to gene relationships by expanding semicolon-delimited gene entries into separate rows. For pathway activity inference, CpG probes were linked to the MSigDB Hallmark gene sets [31] (MSigDB v2023.2) through this annotation, with CpG identifiers remaining the feature units for pathway activity inference where the probes were not averaged to gene level.

#### 2.1.3. Preprocessing and Segmentation of Copy-Number Alteration

We received the CNA data with 413,362 observations or alteration sites across 784 samples, including 709 OAC and 74 BO samples with no normal phenotype samples. The data files consisted of sample id along with chromosome, start, end, total copy number, and minor copy-number details. The CNA data were evaluated for missing values; 24,986 duplicate entries were identified and removed, leaving 388,376 observations. No missing values, negative copy number values, or inconsistencies between minor and total copy numbers were identified.

The $\log_{2}$ ratios of the total copy number relative to the diploid copy number were calculated as log_2_(Total_CN/2) and subsequently normalised by dividing by (1−NormalContamination) to adjust for the estimated normal cell contamination fraction derived from the segmentation statistics of each sample. No samples were excluded on the basis of tumour purity; normalisation was applied to reduce the influence of non-tumour cell contamination on copy number calls. To identify regions with consistent changes in copy number, we then performed segmentation on normalised $\log_{2}$ ratios using circular binary segmentation (CBS) from the DNAcopy library [32] (Version 1.87.0). Here, a 500 kb smooth region parameter was set to reduce the noise along with a minimum segment width of 5 probes to ensure that meaningful segments were retained. Ultimately, the segmented copy-number data were mapped to the genes using GenomicRanges [33] (version 1.54) and biomaRt [34] (version 2.58), with each gene assigned the mean segment value across all overlapping segments, producing a gene-level CNA matrix across samples for integration.

### 2.2. Multi-Omics Data Integration and Feature Selection

To uncover shared molecular mechanisms and dysregulated pathways, we performed multi-omics factor analysis (MOFA2) [6] (version 1.12), a dimensionality reduction technique capable of learning latent factors across heterogeneous data modalities. In total, 231 samples had paired RNA-seq and DNA methylation data (199 OAC, 20 Barrett’s oesophagus, 12 normal tissue), of which 219 additionally had matched CNA data; the 12 normal tissue samples were absent from CNA profiling as copy-number analysis was restricted to tumour tissue. Matched multi-omics data (231 RNA-seq and methylation samples; 219 CNA samples) were pre-filtered for highly variable features (HVFs) using variance percentile cutoffs (RNA-seq: top 70th percentile, minimum variance threshold of 0.50; DNA methylation CpGs: top 30th percentile; CNA: top 50th percentile), followed by z-score scaling on each omics type to ensure data comparability.

The default MOFA2 training and optimisation parameters were used for model training. These included the Gaussian likelihood on all the omics views with varying numbers of latent factors or multi-omics factors (MOFs) (10, 15, 20), spike-and-slab priors on factors and weights, a fast convergence mode, and a maximum of 1000 training iterations. The reason for using varying numbers of MOFs was to optimise and select MOFs based on variance explained across bulk RNA-seq, methylation and CNA data.

Given the phenotypic class imbalance within the OCCAMS dataset, with OAC being the majority class, and the observation that MOFs capture variance relevant to biological differences between phenotypes, we then applied the Kruskal–Wallis test [35] to confirm that the selected MOFs were not biased towards the overrepresented tumour type. From the MOFs considered significant, once identified, we then performed feature selection within each omics view, focusing on features with shared variance across the omics views. The key drivers (genes, CpG, CNA regions) were identified by applying specific absolute weight thresholds to each omics layer (RNA: 0.1; methylation: 0.15; CNA: 0.25).

Additionally, we conducted two levels of pathway enrichment analysis on the selected MOFs: factor-level enrichment, which uses the absolute MOFs loadings across the methylation, RNA, and CNA layers to determine the top pathways that contribute to each of the selected MOFs. We then performed a feature-level analysis, projecting pathway activity across phenotypic groups (normal, BO, and OAC) and multi-omics layers based on the weights of each feature that contributed to the chosen MOFs. To evaluate coordinated changes between phenotypes, the mean pathway activity was calculated for each phenotype and modality. Using a two-tier approach, we were further able to determine the main pathways causing MOF variations, as well as the shift across the disease state taken by each pathway as it progressed from normal to Barrett’s to OAC.

### 2.3. Pathway Activity Inference from a Single- and Multi-Omics Perspective

To characterise multi-layered dysregulation of biological processes, we then proceeded with performing pathway activity inference (PAI) with a single- and multi-omics perspective using both known and novel machine learning and deep learning approaches. We utilised a custom pathway-activity autoencoder (PAAE) [15] using Python to infer pathway-level signals from RNA, methylation (both CpG sites with respective annotated genes), and CNA data using Hallmark gene sets [31]. PAAE enabled us to extract meaningful pathway activity representations from single- and multi-omics data using deep learning to capture intricate, nonlinear interaction. To enhance the interpretability of these learned representations, in parallel, we applied the widely used built-in gene set variation analysis (GSVA) function via the decouplr [13] (version 2.8.0) package in R to determine pathway activities in similar input data. This additional approach allowed us to extract biologically significant pathways and pathway-specific features.

To infer pathway activity, we acquired a lower-dimensional representation of various biological processes driving each omics layer using the (PAAE) framework. Since the methods required a gene-level matrix as input, for single-omics PAI, we utilised the HVFs, whereas for multi-omics PAI, pathway activity inference was performed independently per omics layer using the post-MOFA normalised features from the RNA, methylation, and CNA views, producing three layer-specific pathway activity score matrices across the same 50 Hallmark gene sets, each prefixed by omics layer of origin (rna_, meth_, cna_) and subsequently concatenated column-wise into the combined multi-omics feature matrix (231 samples, 143 features). To ensure biological interpretability, the model drew on prior knowledge of gene–pathway interactions by learning latent representations where each gene corresponded to a specific biological pathway. By transforming the input data in an unsupervised way, the framework incorporated pathway-specific activity while optimising the reconstruction of the original gene expression profiles. These inferred scores from both methods allowed us to extract valuable pathway activity representations from both the single- and multi-omics data. It also provided a clear and interpretable overview of the pathway dynamics across omics layers, which we then used for further downstream analyses.

### 2.4. Characterising Disease Associated Heterogeneity via Inferred Pathway Activity

To investigate the potential of pathway activity inference in characterising disease-associated molecular states, we evaluated pathway scores derived using both PAAE and GSVA in single- and multi-omics data. We used tumour differentiation grade to define Molecular-State1 (well-differentiated, well-to-moderate and moderately differentiated tumours) and Molecular-State2 (moderate-to-poor and poorly differentiated tumours), in accordance with standard oesophageal cancer classification guidelines [36]. Although dysplasia and tumour differentiation may represent related stages along the disease continuum, tumour grade was selected because it provides an ordinal measure of disease severity within established OAC and offered greater prognostic separation in this cohort. A further limitation arises from the clinical assignment of tumour grade, which is based on the highest-grade component present within a specimen. As molecular profiling was performed on bulk tissue, samples may contain mixtures of differentiation states that are not fully captured by a single pathological label. This may introduce noise into grade-associated analyses, although such effects would be expected to attenuate rather than artificially strengthen the observed molecular associations. Samples with indeterminate or unlabelled tumour grades were assigned Unknown, excluded from model training, and reserved as a set held-out for subsequent molecular state projection.

In order to comprehensively evaluate the contribution of various omics modalities and integration methodologies, we applied single-omics models based on HVFs and multi-omics models derived from MOFA-selected features independently to PAAE- and GSVA-based pathway activity scores. The features were z-score normalised using parameters obtained from the training cohort by stratified 80/20 training–test split which was employed to assess stability of state assignment.

To identify a subset of pathways that best distinguished the Molecular-State1 and Molecular-State2 labels, recursive feature elimination [37] was performed. This was followed by elastic net regression using the glmnet package [38] (version 4.1-8), a supervised classification framework that was used to learn pathway-based decision boundaries. Elastic net regression models were trained using five-fold cross-validation repeated 10 times, with hyperparameter tuning implemented to optimise the area under the ROC curve (AUC). ROC analysis and classification measures, such as accuracy, precision, recall, and F1 score, were used to evaluate the model’s performance in the independent test cohort.

Following that, the top-performing single- and multi-omics models were used to project the Unknown samples onto the pathway-defined molecular state space and assigned to the closest molecular state based on pathway activity profiles. The molecular state assignments were then visualised and combined with clinical metadata to later evaluate molecular-state-based survival difference.

### 2.5. Model Interpretability

We then used Shapley Additive Explanations (SHAP) [39] to evaluate the contribution of a pathway to state separation in the best-performing omics combination models using the Interpretable Machine Learning (iml) package [40] (version 0.11.1) in R. To quantify local feature attributions, SHAP values were calculated for each sample, and the final elastic net classifiers trained on MOFA-selected multi-omics pathway activity scores were integrated into a model-agnostic state differentiation framework. To assess the marginal contribution of each pathway to the probability of Molecular-State2 assignment, Shapley values were calculated for each feature–sample pair using Monte Carlo sampling (100 permutations). The absolute SHAP values for each sample were then averaged to estimate the significance of the global pathway, and the pathways with the highest rankings were kept for further analysis and visualisation.

### 2.6. Survival Analysis

Survival differences across molecular states were also evaluated as supportive evidence of biological relevance. Using survival [41] (Version 3.6-3) and survminer [42] (version 0.4.9) R packages, a Kaplan–Meier survival analysis was performed on samples classified as Molecular-State2 and Molecular-State1. Deceased patients were classified as events, and the survival time was calculated as the number of days until death or the last follow-up. Log-rank and Peto–Peto tests [43] were used to assess and estimate disease-state-specific survival difference through survival curves. Curves were displayed with the state tables, annotated time points, and confidence intervals, offering a quantitative evaluation of the multi-omics pathway-activity-based classifier performance.

## 3. Results

### 3.1. Overview of the Pathway-Centric Multi-Omics Integration Framework

**Figure 1:**
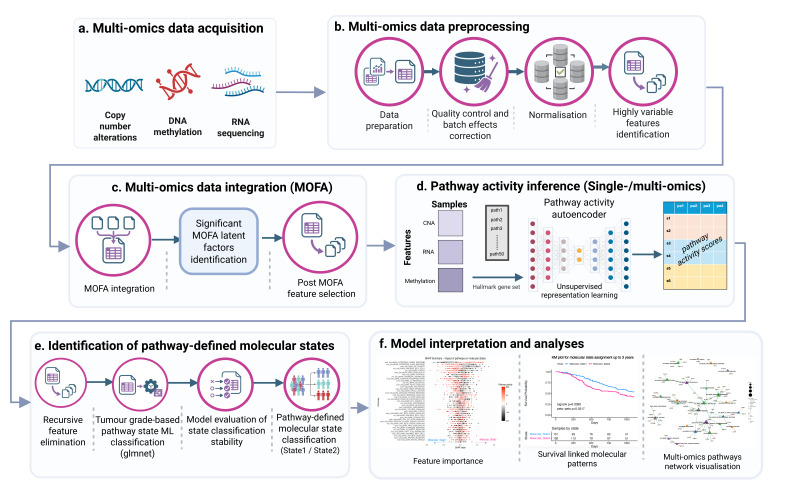
Schematic representation of a pathway-centric multi-omics integration framework. (**a**) Acquisition of multi-omics data acquisition from OCCAMS consortium consisted of copy-number alterations, DNA methylation and bulk RNA-sequencing files. (**b**) Omics layer specific preprocessing involved quality control, batch effects evaluation/correction, normalisation and highly variable features identification. (**c**) For multi-omics data integration, MOFA framework was applied to identify significant features from relevant MOFA latent factors displaying covariance across omics layers. (**d**) Pathway activity inference was performed with both single-omics and integrated multi-omics features. (**e**) To identify predictive features and stratify patients into risk groups, pathway-activity-based risk stratification modelling was performed via recursive feature elimination followed by machine learning predictive model construction. (**f**) Lastly, for interpretation of the pathway-activity-based ML-model results and identification of key pathways and biomarkers, a set of analyses including feature importance based on SHAP values, patient survival assessment and network visualisation were carried out utilising the prognostic features. The network visualisation shown here is a representative excerpt of the full MOFA-PAAE tripartite gene–pathway–omics network presented at full resolution in Figure 3d, where node and edge encodings are described in full. The figure was created in BioRender. Tsoka, S. (2026) https://BioRender.com/10u2wtt (accessed on 12 March 2026).

We developed a pathway-centric multi-omics integration framework that combines latent factor modelling with pathway activity inference and interpretability analysis, presented in Figure 1. The framework is designed to integrate heterogeneous molecular layers while retaining biological interpretability at the pathway level and enabling patient-specific characterisation of molecular states.

The framework takes matched transcriptomic, DNA methylation, and copy-number alteration (CNA) data as input. These data are first integrated using the intermediate integration strategy multi-omics factor analysis (MOFA) to capture shared and modality-specific sources of variation across samples in a low-dimensional latent space. This step yields a unified multi-omics representation that accounts for differences in scale, sparsity, and signal contribution across data types and is used as an intermediate layer for downstream biological abstraction rather than direct interpretation.

To enable biologically interpretable inference, latent representations are decoded to pathway-level activity states using pathway activity modelling based on pathway-activity autoencoders (PAAEs) [15]. This step integrates signals across molecular layers to infer coordinated pathway programs at the patient level, allowing pathway activity to reflect combined contributions from transcriptional, epigenetic and structural alterations. By operating at the pathway level, the framework reduces dimensionality while preserving mechanistic relevance and allowing direct comparison between samples and disease states [44].

The resulting pathway activity states are then used for multiple downstream analyses within a unified framework as shown in Figure 1. These include (i) identification of coordinated molecular programs across omics layers, (ii) organisation of samples along disease-associated molecular states to examine progression-related transitions, (iii) network-informed interpretability analysis to relate pathway activity to gene-level contributions across data types, and (iv) evaluation of biological and survival-associated molecular patterns in addition to pathway-based state classification. Importantly, all analyses are performed on the same integrated pathway representation, ensuring consistency across discovery, interpretation, and evaluation steps.

Together, this framework provides a coherent and interpretable strategy for integrating multi-omics data, enabling the study of disease-associated molecular organisation at the pathway level. In the following sections, we apply this framework to the OCCAMS cohort consisting of Barrett’s oesophagus (BO) and oesophageal adenocarcinoma (OAC) samples, to examine how coordinated pathway activity across molecular layers resolves disease-associated latent structure and shapes molecular states along the normal–Barrett’s–adenocarcinoma continuum.

### 3.2. Multi-Omics Factor Analysis Captures Disease-Associated Latent Structure

**Figure 2:**
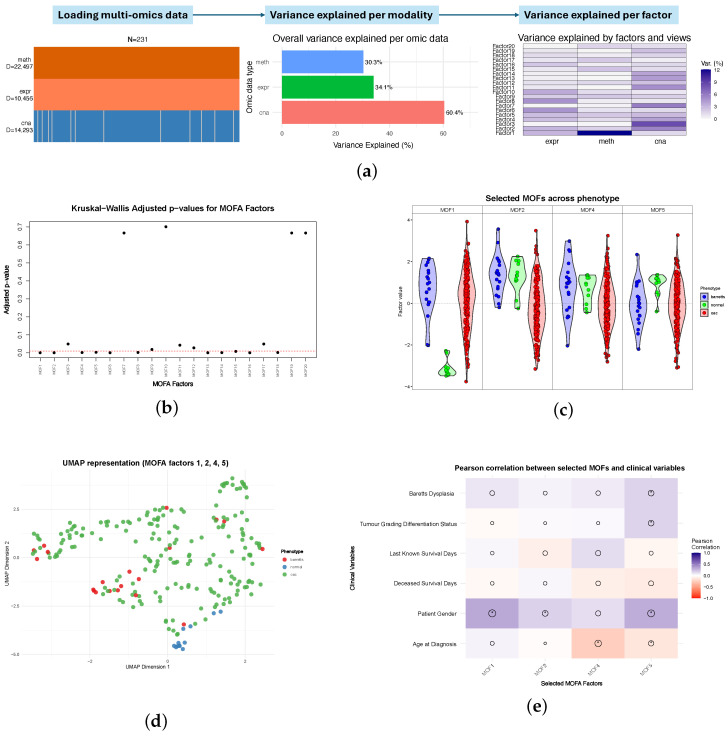
This schematic representation depicts the chronological steps involved in the integration and exploration of multi-omics data: (**a**) After loading the various-modality dataset, the MOFA model reduces the multi-omics data to 20 latent factors. This procedure evaluates each modality and its associated factors’ contribution to variance explanation. The summary and layers of the multi-omics dataset are shown on the left then follows the total variance explained by each modality in the middle and the percentage of variance explained by each individual factor on the right. (**b**) MOF-1, 2, 4, and 5 were found to be significantly associated with phenotype highlighting their relevance for downstream analysis, with the red dashed line indicating the significance threshold (adjusted *p* = 0.01), (**c**) Violin plots illustrating the distribution of these factors across phenotypic groups, supporting the robustness of MOF selection, followed by (**d**) UMAP projection revealing clustering by phenotype and capturing shared variance across omics layers. (**e**) Clinical associations of MOF-5 are summarised, demonstrating its relevance to sex, tumour grade, and adjacent microscopic dysplasia, with asterisks denoting statistically significant Pearson correlations (*p* < 0.05).

MOFA-based integration was performed to explore the molecular mechanisms driving the transition from normal to Barrett’s oesophagus (BO) to oesophageal adenocarcinoma (OAC), and identify multi-omics latent factors (MOFs) explaining coordinated variance between the omics layers. We applied MOFA to integrate bulk RNA-sequencing, DNA methylation, and copy-number alteration (CNA) data from the OCCAMS cohort, capturing tumour heterogeneity beyond single-omics analyses. For the RNA, methylation, and CNA layers, data preprocessing, quality control, and batch evaluation analyses were performed methodically; the technical and biological batch evaluation are shown in Supplementary Figure S1. Trained on 231 matched samples from the OCCAMS consortium, our MOFA model with 20 MOFs provided the total variance explained across the features of the multi-omics layers, as shown in Figure 2a. Having the highest overall variance of 60.4%, CNA explained most of the molecular heterogeneity, followed by the mRNA expression (34.1%) and methylated layers (30.3%) respectively. Methylation contributed the most to MOF-1 with 12.1% variance followed by mRNA expression and CNAs, as shown in Figure 2a.

To identify MOFs displaying significant association with disease state and selected clinical covariates, the Kruskal–Wallis test showed that MOF-1 ($p=1.5\times 10^{-8}$), MOF-2 ($p=3.2\times 10^{-9}$), MOF-4 ($p=4.9\times 10^{-4}$), and MOF-5 ($p=1.4\times 10^{-3}$) had a significant association with the phenotype in Figure 2b. The distinct distribution between the phenotypic groups was further revealed via a violin plot, as shown in Figure 2c, while the UMAP projections in Figure 2d capture the shared variance across the omics layers and highlighted the clear clustering by phenotype, supporting the robustness and biological relevance of these factors. We further identified that MOF-5 presented a strong positive correlation with male sex (Pearson r = 0.303, *p* < 0.001), as well as moderate positive associations with tumour grade (r = 0.158, *p* = 0.016) and adjacent dysplasia (r = 0.162, *p* = 0.014), as shown in Figure 2e, but relatively weak associations with survival results or age at diagnosis. This demonstrated that rather than capturing overall survival, MOF-5 captured molecular variation associated with sex-specific and pathological characteristics. These results collectively suggested that the chosen MOFs indicate disease-relevant latent structures, capturing clinically relevant signals as well as phenotype-related genetic variation, hence further supporting their relevance for downstream feature extraction and inferred pathway activity.

### 3.3. Pathway Activity Modelling Resolves Coordinated Molecular Programs

**Figure 3:**
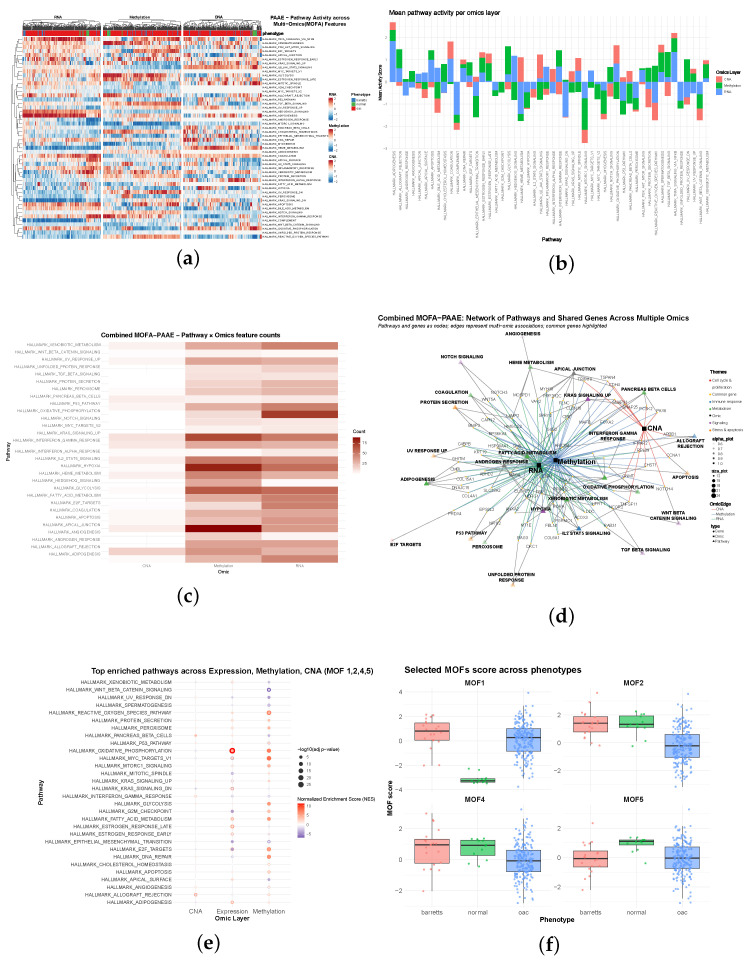
Multi-omics pathway activity analysis in Barrett-associated oesophageal adenocarcinoma (OAC). (**a**) Heatmap of top pathways across normal, Barrett’s oesophagus, and OAC, showing mean pathway activity inferred from MOFA-derived features using PAAE (red, activated; blue, suppressed). (**b**) Mean pathway activity by omics layer, showing layer-specific contributions to Hallmark pathways. (**c**) Combined MOFA–PAAE feature counts per pathway across omics layers, highlighting dominant multi-omics contributions. (**d**) MOFA-PAAE tripartite network linking genes (circles), pathways (triangles), and omics layers (squares: RNA, CNA, methylation). Node size reflects degree centrality; node colour denotes functional pathway class (red = cell cycle and proliferation; blue = immune response; green = metabolism; orange = stress and apoptosis; purple = signalling), with yellow nodes indicating genes shared across two or more omics layers. Node colour intensity (alpha) scales with MOFA feature contribution strength, such that lower-contribution nodes appear in a paler shade of their assigned category colour. Edges are coloured by omics source (RNA green, CNA red, methylation blue) and are undirected, representing pathway–gene co-membership rather than regulatory directionality; edge weight reflects MOFA feature contribution strength. The complete list of hub genes and pathway assignments across omics layers is provided in Supplementary Table S2. (**e**) Dot plot of selected MOFs (1, 2, 4, 5) showing Hallmark pathway enrichment across CNA, expression, and methylation layers. Colour indicates NES; dot size represents significance (–log_10_ adj. *p*-value). (**f**) Latent factor variation across disease stages, highlighting phenotype-specific MOF patterns and associated pathway activity.

To explore the molecular mechanisms behind disease progression, pathway activity inference was performed using deep learning-based PAAE on MOFA-derived multi-omics features, which translated latent molecular variations into pathway-level programs. In parallel, gene set variation analysis (GSVA) and single-omics-level HVFs were compared to PAAE. Here, we focus on the MOFA-PAAE findings, which combine the RNA, methylation, and CNA layers to capture integrated nonlinear multi-layer pathway mechanisms. The most robust biological signals are highlighted by pathways with the highest number of contributing genes or features. These include the metabolic reprogramming processes (oxidative phosphorylation, glycolysis, fatty acid metabolism, peroxisome, haem metabolism, xenobiotic metabolism), proliferation and cell-cycle programs (E2F targets, MYC targets V2, G2M checkpoint, P53 pathway), immune and stress-response processes (interferon α and γ Response, allograft rejection, unfolded protein response, UV response) and structural and oncogenic signalling (apical Junction, notch signalling, Wnt β-Catenin, hedgehog signalling, TGF-β signalling, KRAS signalling up, angiogenesis) as shown in Figure 3a,b.

The fact that these multi-omics pathway programs closely resemble the molecular patterns captured by MOF-5 and that have been linked to male sex, tumour grade, and adjacent dysplasia supports the idea that MOF-5 reflects coordinated immunological, metabolic, and proliferative processes. Although inference of pathway activity was also carried out by GSVA and a single-omics HVFs perspective, MOFA-PAAE was superior by combining multilayered data to identify the most reliable biologically significant oncogenic pathways, which was further reflected by mapping molecular transition across disease-associated states’ output.

To better illustrate the multi-layered relationship of genes, pathways, and omics layers presented in Figure 3c, we constructed a tripartite multi-omics network that allowed visualisation of the co-regulation of the inter- and intra-pathways as well as cross-layer integration. Four key functional modules such as metabolic, immune–inflammatory, stress–survival and signal transduction clusters are highlighted in this network as depicted in Figure 3d, along with hub genes and pathways that have the highest connectivity. A metabolic module linked glycolysis, hypoxia, fatty acid metabolism, and oxidative phosphorylation, connected via key genes such as LDHA, PGK1, and RRAGD. In contrast, a central immune–inflammatory module, including interferon responses, allograft rejection, and IL2-STAT5 signalling, centred on IFNAR2, RORA and NDRG1. Stress–survival module bridged apoptosis, P53, and unfolded protein response through CASP7 and NDRG1. Finally, signal transduction programs such as KRAS, WNT/β-Catenin, notch, and TGF-β converged through NOTCH4 and NCOR2.

The key observations of the network further point to how these modules are functionally organised. Multiple pathway clusters are linked by several hub genes, emphasising their function in regulating various multi-layered biological processes. While CASP7 and LAMP2 dominate stress–survival signalling with contributions across RNA and methylation layers, LDHA1, NDRG13, and ELOVL5 connect metabolic and immunological pathways. Signal transduction hubs, such as MAFB, NCOR2, and NOTCH4, combine signals from several pathways. It should be noted that some genes, including NDRG1, ELOVL5 and SMS, function as connectors between modules, connecting immunological, metabolic, and stress-adaptive programs and suggesting coordinated regulation of many biological processes. Similarly, hub pathways that reflect the main axes of molecular dysregulation were identified as hypoxia, androgen response, xenobiotic metabolism, and glycolysis. In addition to highlighting the similar molecular architecture that underlies pathway activity, this integrative method also demonstrates how multi-omics interactions regulate immunological, metabolic, proliferative, and stress-adaptive activities.

In parallel, for validation of our pathway activity inference results, we performed a MOFA-integrated pathway enrichment analysis. Here, the absolute loadings of the selected MOFs were used to highlight the coordinated molecular features (pathways and associated genes) across the multi-omics layers, along with providing underlying disease-associated molecular variations. In the expression, methylation and CNA layers, consistent patterns were identified by enrichment of strongly related pathways (adjusted *p* < 0.05) as shown in Figure 3e. Initially, we compared MOFA-derived factor scores between normal, BO, and OAC samples to obtain a general understanding of latent factor dynamics in phenotypic states. The selected MOFA factors captured the molecular variations associated with the disease: Factor 1 was higher in BO, Factor 2 was high in BO and in normal state, and Factor 4 was similarly enriched in BO and normal, as shown in Figure 3f. These phenotypic-based pathway dynamics were further explored after mapping molecular transitions across histological states to explore tumour heterogeneity and understand cross-omics coordination between phenotype and histology.

### 3.4. Mapping Molecular Transitions Across Disease-Associated States

The pathway activity profiles were further used to characterise disease-associated heterogeneity by identifying and assigning samples to pathway-defined molecular states and connecting them with tumour differentiation grades. Since survival in our cohort was not significantly differentiated by histological dysplasia alone (log-rank *p* = 0.69), the shortcomings of morphological evaluation were highlighted. Therefore, in accordance with standard oesophageal cancer grading guidelines [36], the sample grouping based on histologically supported tumour grading was used, as it showed marginal significance of log-rank *p* = 0.05. To define the molecular states associated with the pathway, we treated well-differentiated, well-to-moderate and moderately differentiated samples as Molecular-State1 (*n* = 76) and moderate-to-poor and poorly differentiated tumours as Molecular-State2 (*n* = 105). Samples with indeterminate or unlabelled tumour grades were assigned Unknown and were not included in the state definition; following model training, these samples were projected onto the pathway-defined molecular state space, indicating an approximately equal distribution of ambiguous-grade tumours across the two molecular states. To evaluate the efficiency of the pathway activity score in distinguishing disease-associated molecular states, we used PAAE- and GSVA-based pathway scores obtained from both single-omics (HVF) and multi-omics (post-MOFA features) perspectives.

Across all comparisons, PAAE-based models trained on scores obtained from post-MOFA multi-omics features outperformed those trained on PAAE single-omics HVF features or GSVA-based scores. The complete performance findings are included in Supplementary Table S1, while Table 1 summarises the overall accuracy of pathway-activity-based disease assignment in various combinations of omics layers. The role of methylation-driven characteristics in defining disease-associated heterogeneity was demonstrated by the best results obtained with PAAE-MOFA-integrated methylation features alone (accuracy = 0.81), subsequently followed by RNA + methylation (0.78) and RNA + CNA (0.77). When all three omics were integrated, the accuracy stayed strong (0.75), confirming the stability of the PAAE-MOFA framework in a multi-omics setup. However, HVF-derived PAAE models, on the other hand, did not capture the same pathway signals between molecular layers and only produced moderate performance (0.65–0.74).

In the independent test set (*n* = 36), the primary combined MOFA-PAAE model achieved an accuracy of 0.75 and AUC of 0.77, as shown in Table 1. Although the methylation-only model yielded the highest individual accuracy (0.81), the combined three-omics model was retained for downstream analyses because the primary objective of the framework was to identify coordinated pathway programs spanning transcriptomic, epigenomic, and copy-number alterations. The integrated model therefore provides a biologically richer representation of disease-associated molecular states than any single-omics layer alone. Molecular-State1 was identified with precision = 0.636, recall = 0.933 and F1 = 0.756, while Molecular-State2 yielded precision = 0.929, recall = 0.619 and F1 = 0.743. The higher recall for Molecular-State1 and the higher precision for Molecular-State2 reflect contrasting classification behaviour across states, where well-differentiated molecular profiles were broadly captured with few missed cases, while poorly differentiated tumour-associated pathway states were identified with greater selectivity. The stratified training–test split maintained consistent class proportions across both sets (training: 42% Molecular-State1, 58% Molecular-State2; test: 42% Molecular-State1, 58% Molecular-State2), and the per-class metrics confirmed that the model captured both molecular states reliably rather than exploiting differences in class frequency.

**Figure 4:**
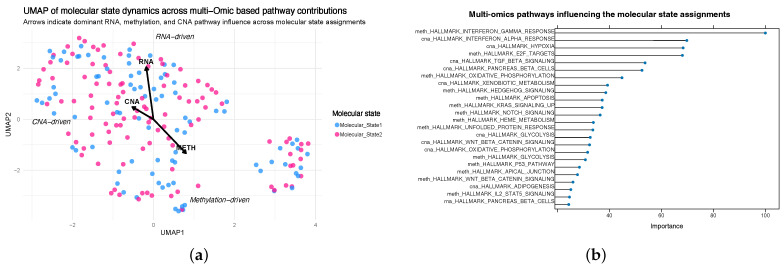
Molecular state assignment based on multi-omics pathway activity using pathway-activity autoencoder (PAAE): (**a**) UMAP projection of multi-omics pathway activity reveals distinct axes corresponding to RNA, methylation, and CNA contributions. RNA-driven pathways predominantly align along the upper vertical axis, whereas methylation-driven pathways opposes that direction. CNA-driven pathways are mostly orthogonal, along a horizontal axis. Along the gradients, there is a great overlap between Molecular_State1 and Molecular_State2, which reflects the integrative regulations across multi-omics layers rather than a single dominant layer. (**b**) Top pathways influencing molecular states are plotted as dots, arranged according to relevance scores obtained within the post-MOFA PAAE model’s output.

Compared to PAAE-MOFA models, GSVA-based modelling performed poorly and was less reliable, even with ideal parameters (RNA only or CNA only, accuracy = 0.68). With several omics combinations falling below 0.35, GSVA-HVF models were especially unstable, highlighting the limited dependability of GSVA in this pathway-defined molecular state modelling context. We visualise the molecular state assignments obtained from the MOFA-PAAE model by projecting samples into a UMAP embedding in Figure 4a, utilising the main pathway activities in the RNA, methylation, and CNA layers. The embedding indicates that although the model identifies clusters of molecular states, the distinction between states is incomplete, highlighting the intrinsic variability of the cohort. This suggests that the molecular states are influenced by a combination of multi-omics signals rather than a single layer. This overlap between states highlights within-cohort heterogeneity and the importance of a multi-omics pathway-activity-based model for accurate assignment of molecular states. It also demonstrates how multi-omics contributions collectively shape the molecular state transition.

The feature importance plot shows projections of the influence of the pathways on the molecular state space in Figure 4b. This highlights methylation, CNA and RNA-derived pathways all have an impact on molecular state assignments, with some pathways having a greater influence than others. In addition, offering a better understanding of the biological programs underlying the pathway influences molecular states. Taken together, our results show that PAAE-obtained pathway activity scores for MOFA-derived features outperform both GSVA- and HVF-based methods, offering a more reliable and consistent framework. Leveraging a deep learning approach, PAAE effectively uses integrative multi-omics features to uncover molecular projections of various disease states by capturing intricate, nonlinear correlations via pathway activity. Furthermore, SHAP-based interpretability assessments are guided by the top pathways identified through the combined PAAE-MOFA model.

#### Key Pathways Emerge from Integrated Omics

In parallel, using post-MOFA pathway enrichment results, we expanded on cross-omics latent factor patterns across disease states by investigating the activity of feature-level pathways in methylation, RNA, and CNA layers. Here, the changes in the CNA-derived pathway referred to the earlier available disease state. In total, 12 Hallmark programs—including oxidative phosphorylation, DNA repair, MYC and E2F targets, KRAS signalling, p53 pathway, fatty acid metabolism, peroxisome, reactive oxygen species (ROS) response, interferon-γ response, apoptosis, and mitotic spindle—were found to be biologically and statistically central to oncogenic, metabolic, and immune reprogramming among significantly enriched pathways.

**Figure 5:**
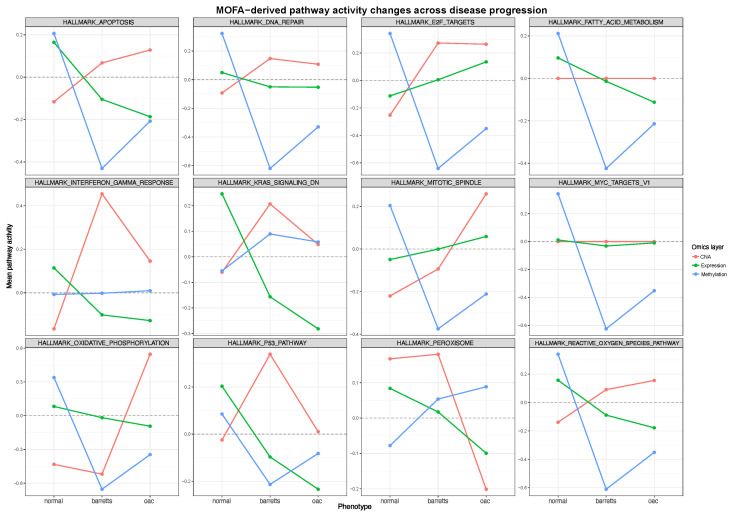
Feature-level mean pathway activity of significantly enriched Hallmark pathways for omics layers is observed in normal, BO, and OAC samples. The dashed horizontal line indicates a mean pathway activity of zero. Pathway shifts across the phenotypes are depicted by lines, where the direction and degree of change are indicated by slope and separation. Across most of the pathways, BO emerges as an intermediate molecular state, exhibiting early epigenetic and structural changes in the immunological, metabolic, DNA damage, and cell-cycle programs that are further enhanced at the transcriptional level in OAC.

Upon visualisation of the pathway shift across the disease states, BO was identified as an intermediate molecular state, as shown in Figure 5, which is consistent with the previous multi-omics description of the transition from BO to OAC [17,45,46]. Indicating partial reprogramming prior to full transcriptional activation, BO shows early structural and epigenetic changes in the mitotic spindle, MYC/E2F, DNA repair, and metabolic programs [47,48]. These pathways are known to play a role in the pathogenesis of OAC, indicating that their early dysregulation in BO offers a molecular foundation for malignant transformation [48]. From normal to BO to OAC, KRAS signalling and E2F targets show sequential increases in CNA and RNA, reflecting increased oncogenic engagement. An epigenetic basis for metabolic rewiring is highlighted by the fact that methylation mainly alters metabolic pathways, such as oxidative phosphorylation, fatty acid metabolism [49], and peroxisome signalling. Although immune-related systems, including interferon γ responses, are activated by CNA early in BO, p53 signalling, ROS response, and apoptosis show cross-omics coordination. These pathways collectively demonstrate a multilayered model of disease progression in which transcriptional dysregulation in OAC can precede and facilitate structural and epigenetic changes in BO.

Expanding on the correlations between MOF5 and male sex, tumour grade, and nearby microscopic dysplasia observed in Figure 2e, we examined the molecular processes that supported this factor. MOF5 was primarily enriched in methylation-associated pathways, with less emphasis on expression pathways and minimal association with CNA. In general, the majority of the enriched pathways in MOF5 were related to metabolism, as depicted in Supplementary Figure S3, where the five most significant pathways were MYC target V1, oxidative phosphorylation, DNA repair, fatty acid metabolism, and glycolysis. However, across the omics layers, oxidative phosphorylation, E2F targets, G2M checkpoint, and pancreas β-cell shared signals across multi-omics layers via pathway enrichment, demonstrating that MOF5 captures integrated, biologically significant mechanisms associated with aggressive disease characteristics. These MOFA-derived pathway enrichment patterns offer a biologically grounded framework for our inferred pathway activity, map molecular transitions across disease states and survival modelling by depicting modality-specific dynamics of key immunologic, metabolic, and oncogenic processes.

### 3.5. Network-Informed Interpretability Links Pathway Programs Across Molecular Layers

**Figure 6:**
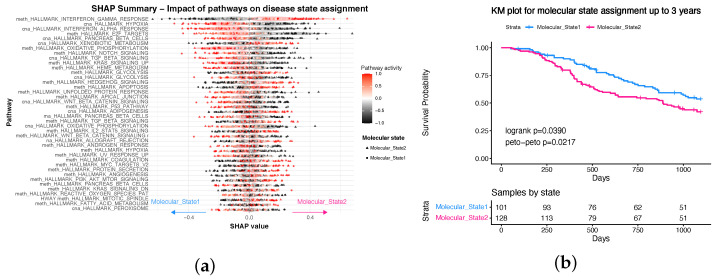
Interpretability of multi-omics pathway-based programs across disease states via PAAE-MOFA: (**a**) The the x-axis indicates the SHAP value, quantifying the impact of each pathway on disease state assignment. Colour encodes pathway activity (red = state2, black = state1), and shape indicates true molecular state label (triangle = Molecular-State2, circle = Molecular-State1). Pathways are ranked by mean absolute SHAP value, highlighting top contributors, including interferon gamma response, hypoxia, E2F targets, TGF-β signalling, and KRAS signalling among others. The spread of state2 and state1 samples across SHAP values illustrates context-dependent, nonlinear interactions, underscoring the ability of PAAE-MOFA to integrate multi-omics signals and capture complex biological determinants of Barrett’s progression to OAC. (**b**) Assignment based on pathway-informed estimation of molecular states shows meaningful early-stage separation of patient-associated molecular states. Three-year survival log-rank *p* = 0.039 and significant Peto–Peto *p* = 0.0217. Despite a moderate cohort (*n* = 231), these results demonstrate the biological relevance of integrated pathway activity scores beyond histology, highlighting their potential for exploring molecular states on multi-omics levels involved in disease progression.

For a multi-layered in-depth interpretability, we concentrated on the findings from the combined MOFA-PAAE multi-omics model for interpretation of pathway contributions to molecular state assignment. To capture context-dependent, nonlinear interactions across different omics layers, global SHAP analysis prioritised pathway programs according to how they contributed to defining the tumour states across the samples. The processes that had the greatest effects were those that were more closely related to immune modulation, cell-cycle regulation, epithelial transformation, and metabolic reprogramming. These processes, shown in Figure 6a, included the interferon gamma response, hypoxia, interferon alpha response, E2F targets, TGF-β signalling, pancreatic β-cell, xenobiotic metabolism, oxidative phosphorylation, notch signalling, KRAS signalling, haem metabolism, glycolysis, hedgehog signalling, and apoptosis.

Interestingly, several of the top genes previously found within the tripartite network presented in Figure 3d—LDHA, NDRG1, ELOVL5, CASP7, LAMP2, NOTCH4, NCOR2, MAFB, IFNAR2, and RORA—also appeared in various SHAP-influential pathways, highlighting the interactions between tumour states defined by pathways and molecular hubs at the network level. For example, the biological alignment of the molecular features underlying Barrett’s development is highlighted by the involvement of CASP7 and IFNAR2 in interferon gamma response and LDHA, NDRG1, PGK1 and RRAGD in hypoxia. In particular, across all multi-omics layers, ELOVL5 was consistently found in various metabolic pathways, including fatty acid metabolism, xenobiotic metabolism, androgen response and peroxisome pathways, as shown in Supplementary Table S2. In oesophageal cancer, ELOVL5 has been identified to play a significant role in metabolic processes due to its established role in fatty acid elongation and lipid metabolism [50], elevated levels of OAC [51], and correlation with tumour mutation burden [52]. In the combined MOFA-PAAE model, ELOVL5 was identified across four Hallmark pathways spanning up to three omics layers (Supplementary Table S2); while this cross-pathway recurrence may partly reflect intrinsic gene set overlap within MSigDB Hallmark collections, ELOVL5 was consistently recovered across multiple molecular layers, appearing in all three omics layers within xenobiotic metabolism and in two omics layers within androgen response, fatty acid metabolism, and peroxisome pathways. This pattern suggests that its repeated identification is not solely attributable to pathway redundancy and is consistent with a recurrent role in OAC-associated metabolic reprogramming. Similarly, NCOR2 has been identified as a patient-specific or rare helper gene involved in the progression from BO to OAC [53]. This SHAP based pathway importance along with network visualisation provides harmonised interpretability by connecting pathway activity to gene-level signals across the omics-layers.

To visualise each pair of sample–pathways, SHAP values (x-axis: influence on pathway defined molecular states), pathway activity (colour: Molecular-State2 red, Molecular-State1 black) and the true molecular state labels (shape: triangle = Molecular-State2, circle = Molecular-State1) were used, as depicted in Figure 6a. The SHAP range is covered by both state2- and state1-risk samples, indicating heterogeneity in the mechanisms involving progression—immune activation being dominant in some individuals, while hypoxia-driven metabolic reprogramming in others—even though increased pathway activity frequently shifted assessments towards elevated molecular state. These findings highlight PAAE-MOFA’s capability to provide mechanistic understanding at both the global and patient-specific levels by capturing nonlinear, multi-omics interactions which conventional methods could overlook.

Significant classification of molecular-state-based disease progression was found in the Kaplan–Meier survival curve of the combined multi-omics MOFA-PAAE model. The model exhibited significant 3-year survival in the early stages (log-rank *p* = 0.039; Peto–Peto *p* = 0.0217) according to the Kaplan–Meier survival analysis shown in Figure 6b, while Supplementary Figure S4 presents the survival results of 10-year (log-rank *p* = 0.1177, Peto–Peto *p* = 0.0394) and 5-year (log-rank *p* = 0.1123, Peto–Peto *p* = 0.0418) survival. Despite a moderate cohort size (*n* = 231), early separation at 3 years provides biological relevance and consistency in the classification of disease states based on this multi-omics pathway-informed strategy by capturing molecular mechanisms that affect progression beyond histological evaluation.

In particular, pathways that were revealed to be significant after MOFA-based pathway enrichment analysis, including interferon gamma response, oxidative phosphorylation, E2F targets, apoptosis, and the p53 pathway, were also among the top SHAP contributors of the combined MOFA-PAAE model. Importantly, these pathways demonstrated elevated activation along the transition from normal state to BO to OAC, which was observed in Figure 5, indicating their association with disease progression. In addition to capturing disease-progression-specific markers, this consistency between methods highlights the ability of the PAAE-MOFA framework to identify biologically significant pathways that replicate the gradual progression across disease states. The pathways that most significantly contributed to the classification of disease states are highlighted by the integration of multi-omics features with SHAP-based interpretability. The pathways include those also involved in metabolic activities, immunological signalling, and cell-cycle regulation in the progression from normal to BO to OAC.

### 3.6. Survival Associations Support Biological Relevance of Integrated Pathway States

**Figure 7:**
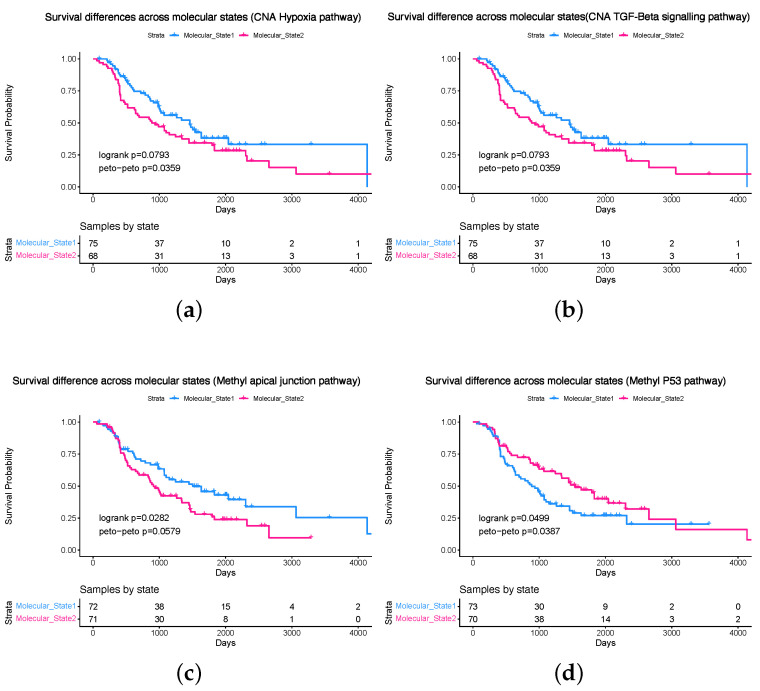

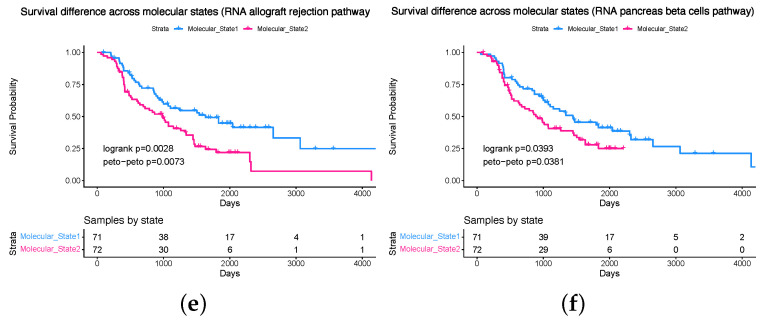
Survival differences across pathway-defined disease states on the combined MOFA-PAAE model: Individual-pathway survival analysis shows significant survival difference across molecular states. (**a**) CNA-mediated hypoxia pathway (logrank *p* = 0.0793, peto–peto *p* = 0.0359); (**b**) CNA-mediated TGF-β signalling pathway (logrank *p* = 0.0793, peto–peto *p* = 0.0359); (**c**) methylation-based apical junction pathway (logrank *p* = 0.0282, peto–peto *p* = 0.0579); (**d**) methylation-based p53 pathway (logrank *p* = 0.0499, peto–peto *p* = 0.0387); (**e**) RNA-regulated allograft rejection pathway (logrank *p* = 0.0028, peto–peto *p* = 0.0073); (**f**) RNA-regulated pancreatic β-cell pathway (logrank *p* = 0.0393, peto–peto *p* = 0.0381).

In addition to assessing survival differences between pathway-defined disease states on the combined MOFA-PAAE model as a whole, we also assessed the significance of classification on individual pathways. To perform individual-pathway-level survival analysis, the complete survival follow-up period was taken into consideration in order to capture long-term outcome associations and sustained pathway-specific effects that go beyond early molecular state separation. Kaplan–Meier analyses provided biological relevance as multiple pathways were identified with significant disease-state-specific survival difference as shown in Figure 7: structurally driven hypoxia (log-rank *p* = 0.0793, Peto–Peto *p* = 0.0359) and TGF-β signalling (log-rank *p* = 0.0793, Peto–Peto *p* = 0.0359); epigenetic-driven apical junction (log-rank *p* = 0.0282, Peto–Peto *p* = 0.0579) and p53 pathway (log-rank *p* = 0.0499, Peto–Peto *p* = 0.0387); transcriptionally regulated allograft rejection (log-rank *p* = 0.0028, Peto–Peto *p* = 0.0073), and pancreas β-cell (log-rank *p* = 0.0393, Peto–Peto *p* = 0.0381).

These findings support our previous SHAP-based analyses by identifying pathways that independently support tumour-grade-associated molecular states in addition to pathway-based state classification. Significantly, the main SHAP-ranked pathways, including hypoxia, TGF-β signalling, and pancreas β-cell, also showed significance for survival, confirming their dual function as significant molecular-state-based drivers. This convergence offers a mechanistic connection between model-driven state-associated signatures and survival-associated molecular patterns, which ultimately highlights how well the PAAE-MOFA framework captures biologically significant features, especially identified to be significant in normal-to-BO-to-OAC progression.

## 4. Discussion

In this study, we introduced a pathway-centric, multi-omics framework to elucidate the molecular mechanisms involved in transitions between disease states. Using the clinically annotated OCCAMS cohort—one of the most comprehensive clinically characterised OAC resources globally—and the integration of RNA, methylation and copy-number alteration (CNA) data, we were able to create a framework centred on pathway-activity inference, demonstrating how structural, epigenetic, and transcriptional alterations were interconnected in driving disease progression. Given the recognised significance of chromosomal instability [54,55], the expanding proof of methylation-dependent immune suppression [56], and transcriptomic dysregulation of metabolic processes involved in the progression from BO to OAC [49], it was essential to use a multi-omics approach.

We were able to capture biologically significant cross-omics signals that shed light on the mechanistic drivers involved in disease state transition by combining multi-omics data using multi-omics factor analysis (MOFA), and incorporating these latent factors with a deep learning-based pathway-activity autoencoder (PAAE) method. This approach, in contrast to traditional single-omics or feature-level approaches, can improve our understanding of disease complexity and allows biologically interpretable characterisation of coordinated molecular organisation across disease progression.

Paired contributions to the underlying disease processes were identified by integrating the multi-omics layers. CNA originally contributed the most to the variance that MOFA was able to capture (Figure 2a); however, further pathway enrichment analysis of multi-omics factors (MOFs) revealed that methylation and RNA layers also made significant contributions to immunological, metabolic and oncogenic processes (Figure 3e) as explored in previous studies [49,57,58]. This variation is probably the result of the complex interaction between structural changes, transcriptional activation, and epigenetic regulation. CNAs might be responsible for providing a favourable environment for subsequent epigenetic or transcriptional changes, a phenomenon observed in various cancers where chromosomal alterations occur prior to functional activation [59,60]. The need for integrated multi-omics models to capture the whole spectrum of molecular drivers was highlighted by our combined PAAE-MOFA model and subsequent interpretability analysis, which further demonstrated that both methylation- and CNA-driven pathways were consistent in distinguishing pathway-defined molecular states.

The shift of pathways along the normal to BO to OAC states further supported our multistep approach. BO was shown to be an intermediate molecular state that was enriched for early disruptions in metabolic pathways including fatty acid metabolism and peroxisome signalling, oxidative phosphorylation, DNA repair and reactive oxygen species (ROS) management (Figure 5). These findings are consistent with recent studies linking metabolic reprogramming and oxidative stress with the development of BO [54]. KRAS signalling, which suggests a temporary early role in disease development, showed a CNA-driven increase between normal state to BO before declining in OAC. This trend is consistent with a study suggesting that KRAS amplifications identify an aggressive subgroup of OAC, although they are not always retained, indicating selective constraints at a specific stage of progression [61]. In contrast, the E2F target pathway provided a steady increase in both the CNA and RNA layers, confirming their notable and stable role in cell-cycle dysregulation throughout development [62]. Metabolic dysregulation, compromised DNA repair, and immunological modulation highlighted that, rather than being a benign state, BO is a molecularly unstable state towards malignant transformation.

Integration of MOFA-derived latent factors in the PAAE framework allowed us to infer the activity of pathways across multi-omics layers [15]. Having the potential to capture complex nonlinear and cross-omics interactions, PAAE outperformed traditional statistical methods by identifying potential pathways and markers in downstream analyses (Table 1). Moreover, survival analysis in our combined PAAE-MOFA model, comprising cross-omics pathways, exhibited a significant assignment of molecular states exhibiting early-stage separation at 3 years of survival with log-rank *p* = 0.039, Peto–Peto *p* = 0.0217 (Figure 6b). This early separation indicates our multi-omics-based pathway-informed molecular state assignments capture biologically significant differences linked to disease progression. The survival analysis based on individual pathways in the combined MOFA-PAAE model identified several factors that were significantly related to patient-defined molecular states, including methylation-driven apical junction and p53; CNA-driven hypoxia and TGF-β signalling; RNA-driven allograft rejection; and pancreas β-cell (Figure 7). However, our findings also revealed a conflict between pathway-defined molecular states and sample-level phenotypic observations.

For example, p53 signalling was lower in SHAP relevance but demonstrated a significant separation between phenotypic states (log-rank *p* < 0.01), indicating that although p53 status has biological relevance in the progression from BO to OAC, as reported by [63,64], the disease-state-based classification model relied more on associated pathways. This finding demonstrates both the benefits and drawbacks of explainable AI techniques. Without necessarily representing biologically or clinically significant patterns, SHAP values alone can highlight characteristics (genes and pathways) that significantly distinguish molecular states. Therefore, we can explore whether relevant molecular states are biologically significant and consistent with disease-associated phenotypes by combining both interpretability with phenotype- or state-associated survival.

Despite these technological advances, it is crucial to acknowledge certain limitations in order to make further improvements to this pathway-activity-inference-based disease-state assignment model. We conducted this analysis by integrating RNA, methylation, and CNA data from 231 matched normal, BO and OAC samples, providing an effective basis to link molecular alterations to patient-defined molecular states. However, larger BO and OAC cohorts will be required to fully verify pathway-level relationships, as the sample size still limits statistical power for subgroup comparisons across disease stages. Moreover, a lack of survival-significant dysplasia labels could not fully reflect the normal to BO to OAC transition, which led us to use tumour grade as an alternative; it should be noted that tumour grade reflects the worst differentiation status observed across the specimen, which may introduce noise in bulk-derived data. Nevertheless, the approximately equal distribution of ambiguous-grade samples across both molecular states suggests that pathway-defined assignment captures molecular heterogeneity that tumour grade alone cannot resolve. Additionally, since we considered working with highly variable features, this feature selection along with omic-specific biases could understate certain RNA- and CNA-based signals while overstating others, e.g., methylation. Finally, although MSigDB Hallmark pathways are helpful for interpretability, they may miss context-specific signalling processes that are better represented by more extensive pathway databases such as Reactome [65] or KEGG [66].

As the framework operates on bulk multi-omics measurements, it does not explicitly resolve contributions from stromal or immune microenvironment components; future integration of immune deconvolution approaches could help disentangle tumour-cell-intrinsic pathway activity from microenvironmental signals, providing a more refined characterisation of the observed molecular states. Furthermore, the multi-omics pathway-centric design of this framework is inherently adaptable to emerging single-cell and spatial transcriptomics modalities, including scRNA-seq, ATAC-seq, and spatial transcriptomics, where pathway activities could be inferred at the resolution of individual cells or spatial spots, enabling finer mapping of coordinated molecular state transitions across tissue architecture. Future work could also evaluate whether the pathway-defined molecular states identified here generalise to Barrett’s oesophagus cohorts with longitudinal follow-up distinguishing progressors from non-progressors, which would further strengthen the clinical translational relevance of the framework. Thus, to improve pathway-based patient-defined molecular state assignments and refine molecular insights, additional research is required to include multi-regional and longitudinal data, along with other multi-omics layers including proteomics, single-cell, and immunological landscapes.

In summary, our study emphasises the significance of a pathway-centric, interpretable multi-omics computational framework for resolving biologically meaningful molecular states from heterogeneous data. By integrating latent factor modelling with pathway-level inference and explainable machine learning, this approach enables predictive signals, functional pathways, and underlying molecular drivers to be systematically aligned across several analytical layers. When applied to the BO and OAC continuum, this framework revealed a strong concordance between pathway-level survival associations and SHAP-based model interpretability, consistently highlighting hypoxia, TGF-β signalling, and pancreatic β-cell as core disease-associated programs. Emphasising their mechanistic relevance, at the gene level, LDHA, NDRG1, ELOVL5, CASP7, LAMP2, NOTCH4, NCOR2, MAFB, IFNAR2, and RORA were identified as shared drivers between SHAP-relevant features and MOFA-based feature-level pathway enrichment (Figure 3d and Table S2). Coordinated pathways and genes highlight the molecular factors that drive the transition from normal to BO to OAC, demonstrating how methylation modifies immunological and metabolic mechanisms, transcriptional processes solidify tumour morphologies, and CNAs provide a favourable environment during disease progression. More broadly, these results establish pathway-centric, interpretable multi-omics integration as a generalisable strategy for mapping molecular transitions underlying disease-associated histopathological states and for prioritising functionally grounded biomarkers for early detection and prevention.

## 5. Conclusions

We reported a pathway-centric, interpretable multi-omics framework that resolved coordinated molecular states across heterogeneous data layers. Our approach offers a scalable and generalisable framework for identifying disease-associated mechanisms and prioritises key multi-omics biomarkers by connecting latent structure with functional pathway activity.

## Figures and Tables

**Table 1:** PAAE- and GSVA-based pathway-defined molecular state classification performance across omics combinations. Values represent accuracy on the held-out test set. Asterisk (*) denotes the best-performing model within each method. The primary model selected for downstream analyses is the combined three-omics PAAE Post-MOFA model (accuracy = 0.75, AUC = 0.77). Complete performance metrics including precision, recall, F1, kappa, and balanced accuracy for all combinations along with molecular state_1 and state_2 are provided in Supplementary Table S1.

Omics Combination	PAAE-Based				GSVA-Based			
	**Post-MOFA**	**AUC**	**Post-HVF**	**AUC**	**Post-MOFA**	**AUC**	**Post-HVF**	**AUC**
All 3 Omics	0.75 *	0.77	0.67	0.67	0.65	0.58	0.59	0.52
RNA Only	0.61	0.54	0.65	0.59	0.68 *	0.62	0.56	0.53
Methylation Only	0.81 *	0.84	0.74	0.80	0.58	0.45	0.59	0.54
CNA Only	0.45	0.60	0.65	0.57	0.68 *	0.70	0.64	0.45
RNA + Methylation	0.78 *	0.82	0.74	0.67	0.59	0.61	0.64	0.60
RNA + CNA	0.77 *	0.78	0.65	0.60	0.68	0.63	0.73	0.66
Methylation + CNA	0.75	0.76	0.78 *	0.83	0.62	0.54	0.32	0.69

## Data Availability

The multi-omics and clinical data analysed in this study were obtained from the Oesophageal Cancer Clinical and Molecular Stratification (OCCAMS) consortium (REC10/H0305/1) and are subject to controlled access. Requests to access the dataset should be directed to the OCCAMS consortium (https://www.occams.org.uk, accessed on 15 April 2026). All custom analytical code is publicly available at https://github.com/sabazaib19/BO-OAC-Pathway-MultiOmics (accessed on 1 June 2026), including step-by-step instructions, a configuration template, and a master pipeline script enabling reproducibility of all analyses and adaptation to new cohorts. The PAAE implementation is available at https://github.com/phcavelar/pathwayae (accessed on 1 June 2026).

## Supplementary Materials

The following supporting information can be downloaded at: https://www.mdpi.com/article/10.3390/cancers18132080/s1, Supplementary figures and tables, along with the list of OCCAMS consortium authors, are provided in separate PDF files. File S1: Figure S1. Assessment of batch effects; Figure S2. Sample overlap across multi-omics OCCAMS cohort; Figure S3. MOFA enriched pathways across multi-omics layers; Figure S4. Kaplan–Meier survival curve of MOFA-PAAE model (complete- and 5-year survival data); Table S1. Molecular state assignment model performance; Table S2. Pathways–genes shared across multiple omics layers in the combined MOFA-PAAE model. File S2: Oesophageal Cancer Clinical and Molecular Stratification (OCCAMS) Consortium.
