# Supplementary material for: Mapping Molecular Transitions in Barrett’s-Associated Oesophageal Adenocarcinoma via Multi-Omics Integration and Pathway Activity Modelling
Source: Cancers (Basel). 2026 Jun 26;18(13):2080. doi: 10.3390/cancers18132080 (PMC13359491; doi:10.3390/cancers18132080)
Supplement: Supplementary file 1 [file cancers-18-02080-s001.zip › Supplementary File S1.pdf]

Supplementary Material File S1

Figure S1: Assessment of batch effects

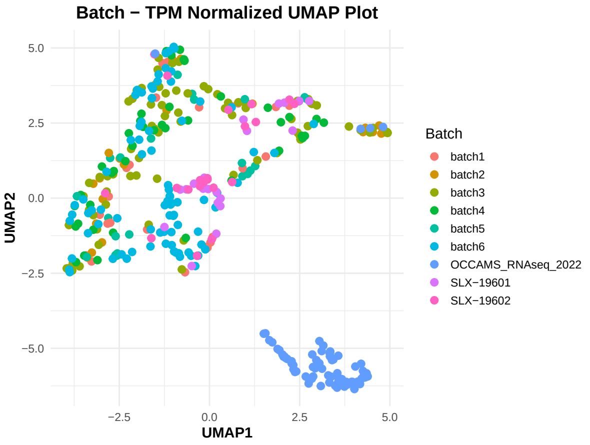

(a)

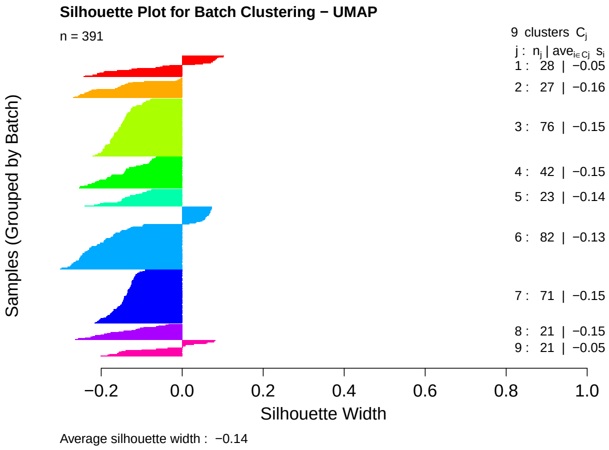

(b)

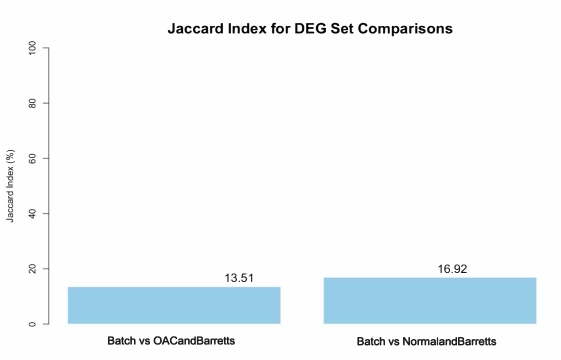

(c)

Figure S1: **Evaluation of potential batch effects: a** UMAP visualisation of the samples revealed a distinct cluster corresponding to a specific batch, prompting further evaluation of batch effects. **b** Technical evaluation using silhouette score analysis revealed most samples having scores near -1, indicating well-mixed data and minimal batch effects. **c** Biological evaluation using the Jaccard index further compared differential expression gene sets across batches. The indices for batch vs DEGenes from oesophageal adenocarcinoma and Barrett's samples (13.51) and batch vs DEGenes from normal and Barrett's samples (16.92) were low, indicating limited influence of batch on phenotype-associated DEGenes. Collectively, these analyses indicate that batch effects are minimal. Therefore, no batch correction was applied.

Figure S2: Sample overlap across multi-omics OCCAMS cohort

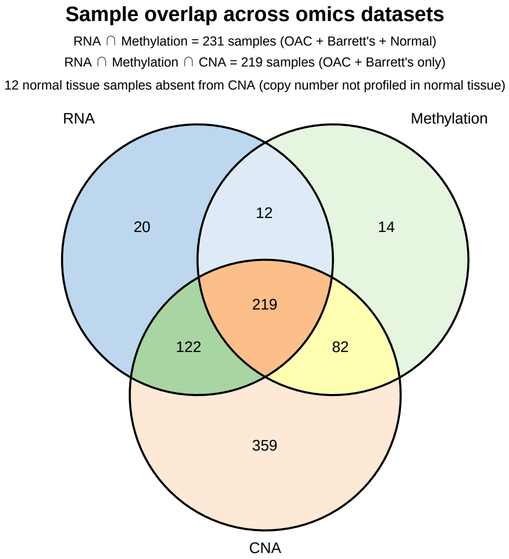

Figure S2: **Sample overlap across RNA-seq, DNA methylation, and CNA datasets from the OCCAMS cohort.** The Venn diagram illustrates the intersection of samples across the three omics modalities. RNA-seq and DNA methylation share 231 matched samples spanning OAC (n = 199), Barrett's oesophagus (n = 20), and normal tissue (n = 12). Of these, 219 samples additionally had matched CNA data; the 12 samples present in RNA-seq and methylation but absent from CNA correspond to normal tissue samples, for which copy number profiling was not available in the OCCAMS cohort. CNA data contains OAC and Barrett's oesophagus samples only.

Figure S3: MOFA enriched pathways across multi-omics layers

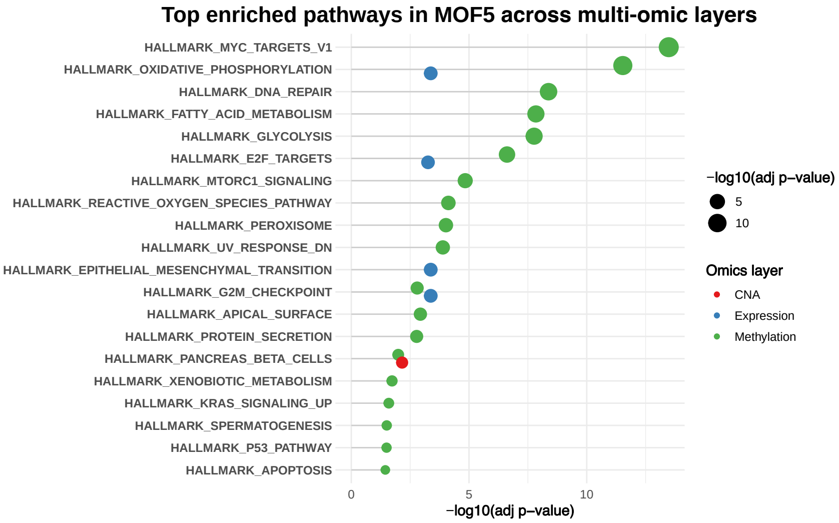

Figure S3: Pathway enrichment analysis of MOF5 revealed a predominance of metabolism-related pathways. The five most significantly enriched pathways were MYC targets V1, oxidative phosphorylation, DNA repair, fatty acid metabolism, and glycolysis.

Figure S4. Kaplan-Meier survival curve of MOFA-PAAE model (Complete- and 5-years survival data)

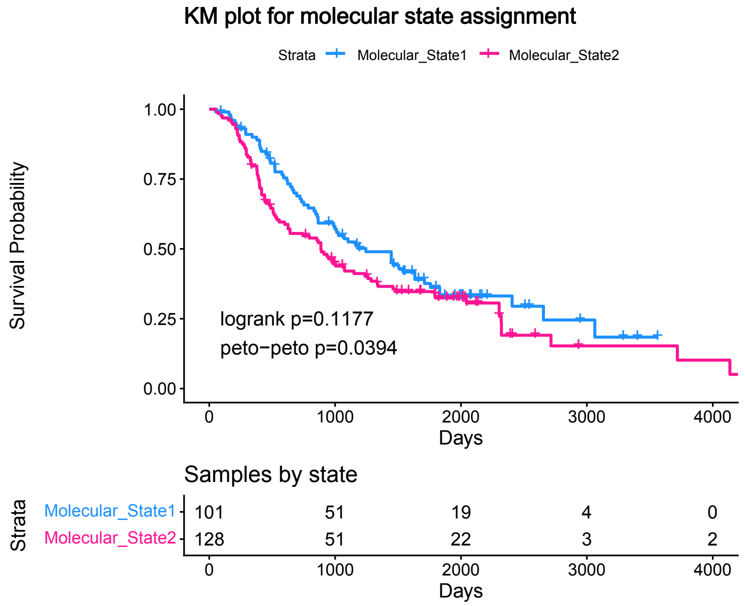

(a)

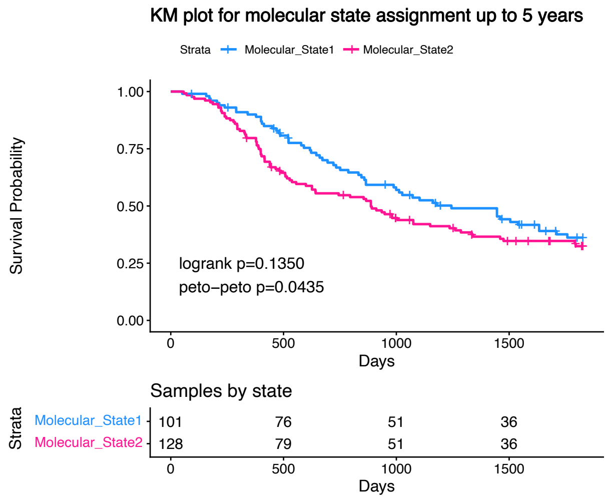

(b)

Figure S4: **Kaplan–Meier survival analysis at 10 and 5 years where statistical significance was assessed using the log-rank and peto–peto tests.** Kaplan–Meier survival curves showing (a) complete and (b) 5-year survival across the indicated groups. Here, the Peto-Peto test revealed significance whereas the log-rank test did not. The difference may be due to increased censoring and reduced statistical power over a prolonged patient follow-up, rather than a loss of underlying biological signals.

Table S1: Molecular state assignment model performance

Table S1: **Molecular state assignment performance metrics (PAAE and GSVA).** Recall (S2) = Sensitivity for Molecular-State2; Recall (S1) = Specificity for Molecular-State1; Precision (S2) = Positive Predictive Value; Precision (S1) = Negative Predictive Value; F1 (S2) and F1 (S1) = harmonic mean of precision and recall for each state respectively.

**Omics Combination**	**Accuracy**	**95% CI**	**p-value**	**Recall (S2)**	**Recall (S1)**	**Kappa**	**Balanced Accuracy**	**Precision (S2)**	**Precision (S1)**	**F1 (S2)**	**F1 (S1)**	**AUC**
**Pathway activity autoencoders (PAAE) - Post-MOFA features**												
All combined	0.75	(0.578, 0.8788)	0.02898	0.619	0.9333	0.5179	0.7762	0.9286	0.6364	0.743	0.756	0.77
RNA only	0.6111	(0.4346, 0.7686)	0.4367	0.7619	0.4	0.1683	0.581	0.64	0.5455	0.696	0.468	0.54
Methylation only	0.8056	(0.6398, 0.9181)	0.004261	0.8095	0.8	0.6038	0.8048	0.85	0.75	0.829	0.774	0.841
CNA only	0.4167	(0.2551, 0.5924)	0.9854	0.619	0.1333	−0.26	0.3762	0.5	0.2	0.553	0.160	0.595
RNA+Methylation	0.7778	(0.6085, 0.8988)	0.01193	0.8095	0.7333	0.5429	0.7714	0.8095	0.7333	0.810	0.733	0.819
RNA+CNA	0.7778	(0.6085, 0.8988)	0.01193	0.8571	0.6667	0.534	0.7619	0.7826	0.7692	0.818	0.716	0.775
Methylation+CNA	0.75	(0.578, 0.8788)	0.02898	0.619	0.9333	0.5179	0.7762	0.9286	0.6364	0.743	0.756	0.762
**Pathway activity autoencoders (PAAE) - Highly variable features**												
All combined	0.6667	(0.4903, 0.8144)	0.2	0.6667	0.6667	0.3271	0.6667	0.7368	0.5882	0.700	0.625	0.673
RNA only	0.6522	(0.4273, 0.8362)	0.1483	0.6667	0.6364	0.303	0.6515	0.6667	0.6364	0.667	0.649	0.591
Methylation only	0.7391	(0.5159, 0.8977)	0.0284	0.6667	0.8182	0.4812	0.7424	0.8	0.6923	0.727	0.750	0.795
CNA only	0.6522	(0.4273, 0.8362)	0.1483	0.5833	0.7273	0.3083	0.6553	0.7	0.6154	0.636	0.664	0.568
RNA+Methylation	0.7391	(0.5159, 0.8977)	0.0284	0.5833	0.9091	0.4851	0.7462	0.875	0.6667	0.700	0.762	0.667
RNA+CNA	0.6522	(0.4273, 0.8362)	0.14826	0.3333	1	0.3235	0.6667	1	0.5789	0.500	0.733	0.598
Methylation+CNA	0.7826	(0.563, 0.9254)	0.009401	0.6667	0.9091	0.5693	0.7879	0.8889	0.7143	0.762	0.800	0.826
**Gene set variation analysis (GSVA) - Post-MOFA features**												
All combined	0.6471	(0.4649, 0.8025)	0.3036	0.7	0.5714	0.2714	0.6357	0.7	0.5714	0.700	0.571	0.575
RNA only	0.6765	(0.4947, 0.8261)	0.19268	0.5	0.9286	0.3909	0.7143	0.9091	0.5652	0.645	0.706	0.621
Methylation only	0.5882	(0.407, 0.7535)	0.5729	0.5	0.7143	0.2013	0.6071	0.7143	0.5	0.588	0.588	0.45
CNA only	0.6765	(0.4947, 0.8261)	0.1927	0.65	0.7143	0.3529	0.6821	0.7647	0.5882	0.703	0.645	0.704
RNA+Methylation	0.5882	(0.407, 0.7535)	0.57294	0.45	0.7857	0.2171	0.6179	0.75	0.5	0.563	0.609	0.611
RNA+CNA	0.6765	(0.4947, 0.8261)	0.1927	0.75	0.5714	0.3249	0.6607	0.7143	0.6154	0.732	0.585	0.629
Methylation+CNA	0.6176	(0.4356, 0.7783)	0.435	0.6	0.6429	0.2353	0.6214	0.7059	0.5294	0.649	0.585	0.536
**Gene set variation analysis (GSVA) - Highly variable features**												
All combined	0.5882	(0.407, 0.7535)	0.5729	0.55	0.6429	0.1849	0.5964	0.6875	0.5	0.611	0.556	0.518
RNA only	0.5588	(0.3789, 0.7281)	0.701697	0.3	0.9286	0.2006	0.6143	0.8571	0.4815	0.444	0.632	0.525
Methylation only	0.5909	(0.3635, 0.7929)	0.2617	0.2727	0.9091	0.1818	0.5909	0.75	0.5556	0.400	0.682	0.537
CNA only	0.6364	(0.4066, 0.828)	0.1431	0.8182	0.4545	0.2727	0.6364	0.6	0.7143	0.692	0.556	0.446
RNA+Methylation	0.6364	(0.4066, 0.828)	0.14314	0.2727	1	0.2727	0.6364	1	0.5789	0.429	0.733	0.603
RNA+CNA	0.7273	(0.4978, 0.8927)	0.02624	0.7273	0.7273	0.4545	0.7273	0.7273	0.7273	0.727	0.727	0.661
Methylation+CNA	0.3182	(0.1386, 0.5487)	0.9738	0.4545	0.1818	−0.3636	0.3182	0.3571	0.25	0.400	0.154	0.694

Table S2: Pathways-Genes shared across multiple omics layers in the combined MOFA-PAAE model.

Pathway	Genes	Omic layers	n
Hallmark xenobiotic metabolism	ABCC2, ACOX3, CBR1, DDC, ELOVL5, FBLN1, FBP1, GNMT, HPRT1, MAOA, PGRMC1	CNA, Methylation, RNA	11
Hallmark androgen response	ABHD2, ARID5B, ELOVL5, KRT19, NDRG1, RPS6KA3, SLC38A2, SMS	Methylation, RNA	8
Hallmark fatty acid metabolism	CBR1, ELOVL5, G0S2, HMGCS2, HSP90AA1, LDHA, MAOA, SMS	Methylation, RNA	8
Hallmark hypoxia	ANXA2, FBP1, LDHA, MT1E, NDRG1, PGK1, RORA, RRAGD	Methylation, RNA	8
Hallmark apical junction	CDH4, CLDN18, FLNC, MYH10, PPP2R2C, TSPAN4, VAV2	CNA, Methylation, RNA	7
Hallmark glycolysis	CHST1, IL13RA1, LDHA, PGK1, PKP2, RRAGD	CNA, Methylation, RNA	6
Hallmark KRAS signalling UP	CPE, G0S2, MAFB, SNAP25, SNAP91, SPRY2	CNA, Methylation, RNA	6
Hallmark adipogenesis	CMBL, COL15A1, COL4A1, DNAJC15, GHITM	Methylation, RNA	5
Hallmark IL2 STAT5 signalling	COL6A1, NDRG1, RORA, RRAGD, TNFSF11	CNA, Methylation, RNA	5
Hallmark pancreas beta cells	FOXA2, MAFB, PAX6, PCSK2	CNA, Methylation, RNA	4
Hallmark allograft rejection	APBB1, IFNAR2, RPL39	CNA, Methylation, RNA	3
Hallmark coagulation	CAPN2, HMGCS2, LAMP2	Methylation, RNA	3
Hallmark heme metabolism	LAMP2, MOSPD1, TRIM10	CNA, Methylation, RNA	3
Hallmark interferon γ response	ARID5B, CASP7, IFNAR2	CNA, Methylation, RNA	3
Hallmark oxidative phosphorylation	CASP7, LDHA, MRPL35	Methylation, RNA	3
Hallmark protein secretion	BNIP3, LAMP2, RPS6KA3	Methylation, RNA	3
Hallmark apoptosis	CASP7, CCNA1	CNA, Methylation, RNA	2
Hallmark notch signalling	NOTCH3, WNT5A	Methylation, RNA	2
Hallmark P53 pathway	EPS8L2, NDRG1	Methylation, RNA	2
Hallmark peroxisome	ELOVL5, NR1I2	Methylation, RNA	2
Hallmark TGF BETA signalling	NCOR2, RAB31	Methylation, RNA	2
Hallmark unfolded protein response	BAG3, DKC1	Methylation, RNA	2
Hallmark UV response UP	C4BPB, MAOA	Methylation, RNA	2
Hallmark WNT-β catenin signalling	NCOR2, NOTCH4	CNA, Methylation, RNA	2
Hallmark angiogenesis	VAV2	Methylation, RNA	1
Hallmark E2F targets	PRDX4	Methylation, RNA	1
