# Supplementary material for: Mapping Molecular Transitions in Barrett’s-Associated Oesophageal Adenocarcinoma via Multi-Omics Integration and Pathway Activity Modelling
Source: Cancers (Basel). 2026 Jun 26;18(13):2080. doi: 10.3390/cancers18132080 (PMC13359491; doi:10.3390/cancers18132080)
Supplement: Supplementary file 1 [file cancers-18-02080-s001.zip › Supplementary File S2.pdf]

File S2. Oesophageal Cancer Clinical and Molecular Stratification (OCCAMS) Consortium:

Rebecca C. Fitzgerald[1], Paul A.W. Edwards[1,2], Nicola Grehan[1,5], Barbara Nutzinger[1], Aisling M Redmond[1], Sujath Abbas[1], Adam Freeman[1], Elizabeth C. Smyth[5], Christopher Jones[1,5], Maria O'Donovan[1,3], Ahmad Miremadi[1,3], Shalini Malhotra[1,3], Monika Tripathi[1,3], Calvin Cheah[1,3], Hannah Coles[1], Matthew Eldridge[2], Maria Secrier[2], Ginny Devonshire[2], Sriganesh Jammula[2], Jim Davies[4], Charles Crichton[4], Nick Carroll[5], Richard H. Hardwick[5], Peter Safranek[5], Andrew Hindmarsh[5], Vijayendran Sujendran[5], Stephen J. Hayes[6,13], Yeng Ang[6,7,26], Andrew Sharrocks[26], Shaun R. Preston[8], Izhar Bagwan[8], Vicki Save[9], Richard J.E. Skipworth[9,20], J. Robert O'Neill[5,9,20], Olga Tucker[10,29], Andrew Beggs[10,25], Philippe Taniere[10], Sonia Puig[10], Gianmarco Contino[10], Timothy J. Underwood[11,12], Ben L. Grace[11], Jesper Lagergren[14,22], James Gossage[14,21], Andrew Davies[14,21], Fuju Chang[14,21], Ula Mahadeva[14], Vicky Goh[21], Francesca D. Ciccarelli[21], Grant Sanders[15], David Chan[15], Ed Cheong[16], Bhaskar Kumar[16], L. Sreedharan[16], Simon L Parsons[17], Irshad Soomro[17], Philip Kaye[17], John Saunders[6,17], Laurence Lovat[18], Rehan Haidry[18], Michael Scott[19], Sharmila Sothi[23], George B. Hanna[27], Christopher J. Peters[27], Krishna Moorthy[27], Anna Grabowska[28], Richard Turkington[30], Damian McManus[30], Helen Coleman[30], Russell D Petty[32], Freddie Bartlett[33], Tom D.L. Crosby[34]

[1]Early Cancer Institute, University of Cambridge, Cambridge, UK
[2]Cancer Research UK Cambridge Institute, University of Cambridge, Cambridge, UK
[3]Department of Histopathology, Addenbrooke's Hospital, Cambridge, UK
[4]Department of Computer Science, University of Oxford, Oxford, UK
[5]Cambridge University Hospitals NHS Foundation Trust, Cambridge, UK
[6]Salford Royal NHS Foundation Trust, Salford, UK
[7]Wigan and Leigh NHS Foundation Trust, Wigan, UK
[8]Royal Surrey County Hospital NHS Foundation Trust, Guildford, UK
[9]Edinburgh Royal Infirmary, Edinburgh, UK
[10]University Hospitals Birmingham NHS Foundation Trust, Birmingham, UK
[11]University Hospital Southampton NHS Foundation Trust, Southampton, UK
[12]Cancer Sciences Division, University of Southampton, Southampton, UK
[13]Faculty of Medical and Human Sciences, University of Manchester, Manchester, UK
[14]Guy's and St Thomas's NHS Foundation Trust, London, UK
[15]Plymouth Hospitals NHS Trust, Plymouth, UK
[16]Norfolk and Norwich University Hospital NHS Foundation Trust, Norwich, UK
[17]Nottingham University Hospitals NHS Trust, Nottingham, UK
[18]University College London, London, UK
[19]Wythenshawe Hospital, Manchester, UK
[20]University of Edinburgh, Edinburgh, UK
[21]King's College London, London, UK
[22]Karolinska Institutet, Stockholm, Sweden
[23]University Hospitals Coventry and Warwickshire NHS Trust, Coventry, UK
[25]Institute of Cancer and Genomic Sciences, University of Birmingham, Birmingham, UK
[26]GI Science Centre, University of Manchester, Manchester, UK
[27]Department of Surgery and Cancer, Imperial College London, London, UK
[28]Queen's Medical Centre, University of Nottingham, Nottingham, UK
[29]Heart of England NHS Foundation Trust, Birmingham, UK
[30]Centre for Cancer Research and Cell Biology, Queen's University Belfast, Belfast, UK
[32]Tayside Cancer Centre, Ninewells Hospital and Medical School, Dundee, UK
[33]Portsmouth Hospitals NHS Trust, Portsmouth, UK
[34]Velindre University NHS Trust, Cardiff, UK
